# Population-level assessment of atlas occipitalization in artificially modified crania from pre-Hispanic Peru

**DOI:** 10.1371/journal.pone.0239600

**Published:** 2020-09-24

**Authors:** Laura N. Pott, Rita M. Austin, Andrea R. Eller, Courtney A. Hofman, Sabrina B. Sholts

**Affiliations:** 1 Department of Anthropology, University of Oklahoma, Norman, Oklahoma, United States of America; 2 Laboratories of Molecular Anthropology and Microbiome Research, University of Oklahoma, Norman, Oklahoma, United States of America; 3 Department of Anthropology, National Museum of Natural History, Smithsonian Institution, Washington, DC, United States of America; The Cyprus Institute, CYPRUS

## Abstract

Atlas occipitalization (AO) is a spinal anomaly, characterized by the fusion of the first cervical vertebra and occipital bone, with a complex etiology that can arise from congenital and environmental causes. AO has been reported in three regions of pre-Hispanic Peru in skeletal remains with artificial cranial modification (ACM), which involves the use of compression devices to permanently alter cranial shape and may have affected the fusion of the atlas and occipital bone. The aims of this study were to gain insights into AO’s etiology by testing correlations between AO and ACM presence/type and geographic region as well as to characterize morphological variation associated with AO. We investigated the geographic distribution of AO and its potential relationship to ACM in a large sample of human crania from eight coastal and highland regions of pre-Hispanic Peru, held at the Smithsonian’s National Museum of Natural History (n = 608, 1300–1500 CE). Eleven cases of AO were observed in three coastal regions—including two previously unreported regions—at an overall frequency of 1.8%. The frequency of AO did not differ significantly between crania with and without ACM, in general or by type, suggesting that ACM is not an etiological factor that influences AO in this sample. AO was observed at a significantly higher rate in the southern coastal region of Arequipa than in any other region. Genetic, dietary, and epidemiological conditions are evaluated as factors possibly shaping the geographic distribution of AO along the central and southern coasts of Peru.

## Introduction

Atlas occipitalization (AO), also termed atlanto-occipital fusion, atlas assimilation, and occipitocervical synostosis, is a spinal anomaly characterized by the partial or complete fusion of the first cervical (C1) vertebra, or atlas, to the occipital bone [1]. AO has been documented in human remains from archaeological sites across the world over the last few millennia [2–12]. AO occurs in around 1% of prehistoric populations [5] and in 0.03–3.6% of contemporary populations [13–15]. AO is often asymptomatic, but when symptoms do appear, they usually do so after the young adult years [16]. Reports of AO in medical literature frequently describe its neurological effects and surgical implications [15, 17–21].

Understanding AO’s symptoms and associated skeletal anomalies is an ongoing challenge in both anthropological and medical research. AO shifts the first mobile segment between the skull and spine to the C1–C2 junction, which can cause physical stress [22]. People with more severe forms of this anomaly may experience headaches and neck pain [16, 23], dizziness and reduced blood flow to the brain [18], numbness in the extremities [16], dislocations of the cervico-occipital joint [23], restricted head and neck mobility [10, 24], sensory disturbances [17], and even death [25]. AO often occurs alongside anomalies such as block vertebrae [3], basilar impression [26], spina bifida of the posterior arch of the atlas [1], fused transverse processes of the atlas [21], size reduction of the foramen magnum [16, 21], and changes in the route of the vertebral artery into the brain [27]. AO may also co-occur with compressive myelopathy [26–28], Klippel-Feil syndrome [29], or torticollis [16, 23].

AO likely results from a complex interplay of factors, so its etiology is still not fully understood [30]. In general, AO is considered to be a congenital condition caused by the failure of the first cervical sclerotome, a vertebral precursor, to divide into cranial and caudal halves during the third week of fetal development [1]. Factors such as nutrition- or disease-related disturbances during development [8, 31] or genetic anomalies [17] may also predispose a fetus toward developing AO. AO has also been linked to traumatic injuries of the cervical vertebrae [11], malnutrition [8], and diseases like osteoarthritis [11], tuberculosis or syphilis [27], and other infections [31].

Isolated instances of AO have been documented in Zoque remains with artificial cranial modification (ACM) from a cave in Chiapas, Mexico [11], as well as in three regions of pre-Hispanic Peru [3, 4] in which ACM was practiced [32]. However, the potential relationship between ACM and AO has not been systematically explored. ACM is a cultural process that applies mechanical stress to permanently alter the shape of an individual’s head [33]. During infancy or early childhood, while the cranial bones are still malleable, adults carers use compression devices to intentionally redirect cranial growth [34]. Between the ages of two and three years old, the cranial bones fuse and these cranial shapes become permanent [33]. Around the world, ACM has been used to imbue head shape with layers of culturally-specific meanings that relate to ethnicity, status, gender, geographic origin, intragroup solidarity, or intergroup distinctions [33–35].

This process of ACM coincides with the postnatal period in which the atlas ossifies and fuses [36]. The atlas approaches its adult size between 4–6 years of age, around the same time that individual parts of the occipital bone complete fusion [36]. Due to the sensitivity of the craniocervical junction [3], this area thus may be particularly susceptible to mechanical stressors like ACM during early stages of skeletal development. As ACM has already been shown to physiologically alter the morphology of other cranial tissues, it could also have some association with AO [37–39]. In addition, because the atlas plays a key role in transmitting the weight of the head to the rest of the vertebral column [1], the restriction of head movement associated with ACM may contribute to morphological changes like AO by changing how the atlas bears this weight. Given that fusion of the atlas with the occipital bone can be localized or extensive, including multiple variations of partial AO that involve some degree of atlanto-occipital articulation [15], it is also possible that ACM could influence the expression of this anomaly even in cases of a congenital origin.

In this study, we investigated the potential relationship between AO and ACM in a large skeletal sample (n = 608) of pre-Hispanic Peruvian remains (1300–1500 CE). We hypothesized that if ACM affects AO, then crania with ACM will have significantly higher frequencies of AO than unmodified crania. The size and geographic diversity of this sample allowed us to test population-level correlations between AO and ACM presence and type, sex, and region. Our aims were to gain insights into AO’s etiology and to characterize broader patterns in geographic and morphological variation in AO across pre-Hispanic Peru.

## Materials and methods

The sample for this study consists of 608 crania held at the Smithsonian Institution’s National Museum of Natural History (NMNH), Washington, D.C. The NMNH holds the largest collection of Peruvian human remains outside of Peru, which was assembled by Aleš Hrdlička in the early 20^th^ century [32]. Ensuring that remains came from pre-Hispanic individuals was more important to Hrdlička than precise provenience, so many individuals were labelled with the name of a valley or a continuously-occupied site [32]. This study therefore focuses on a regional-level comparison of AO frequency across Peru, offering in regional trends what it cannot in site-level specificity or fine-grained chronology. The crania come from sites in eight coastal and highland regions across western and southern Peru (Table 1), and although they have no radiocarbon dates, it is estimated that they date to the late prehistoric period (ca. 1300–1500 CE) based on their preservation and collection context [40; David Hunt, personal communication, August 2020]. In most cases, the crania were not associated with postcranial remains. No permits were required for the described study, which complied with all relevant regulations.

**Table 1:** Geographical distribution of atlas occipitalization (AO).

Region Number	Region	Localities	Without AO		With AO		Total	
			n	%	n	%	n	%
2	La Libertad	Chan Chan, Huanchaco, Pacasmayo, Valley of Chicama	294	100.0	0	0.0	294	48.4
3	Áncash	Copa Chica/Callejón de Huaylas, Macate	2	100.0	0	0.0	2	0.1
4	Lima	Ancón, La Capilla, Chachlacayo, Chancay, Chilca, Huacá-Puná, Pachacamac, Paramonga, Pasamayo, Rimac Valley, San Lorenzo Island, Supé, Zapallar/Puenta Piedra	101	99.0	1	1.0	102	16.8
5	Junín	Caudivilla, La Oroya, Quiterada, Tarmatambo	29	100.0	0	0.0	29	1.6
6	Huancavelica	Acobamba	10	100.0	0	0.0	10	0.6
7	Ica	Coyungo, Nazca Region, Paracas Peninsula	86	95.6	4	4.4	90	14.8
8	Arequipa	Chaviña (Acarí Valley), Lomas	70	92.1	6	7.9	76	12.5
9	Cuzco	Picicalca, San Pedro	5	100.0	0	0.00	5	0.3
	Total	-	597	98.2	11	1.8	608	100.0

**Fig 1:**
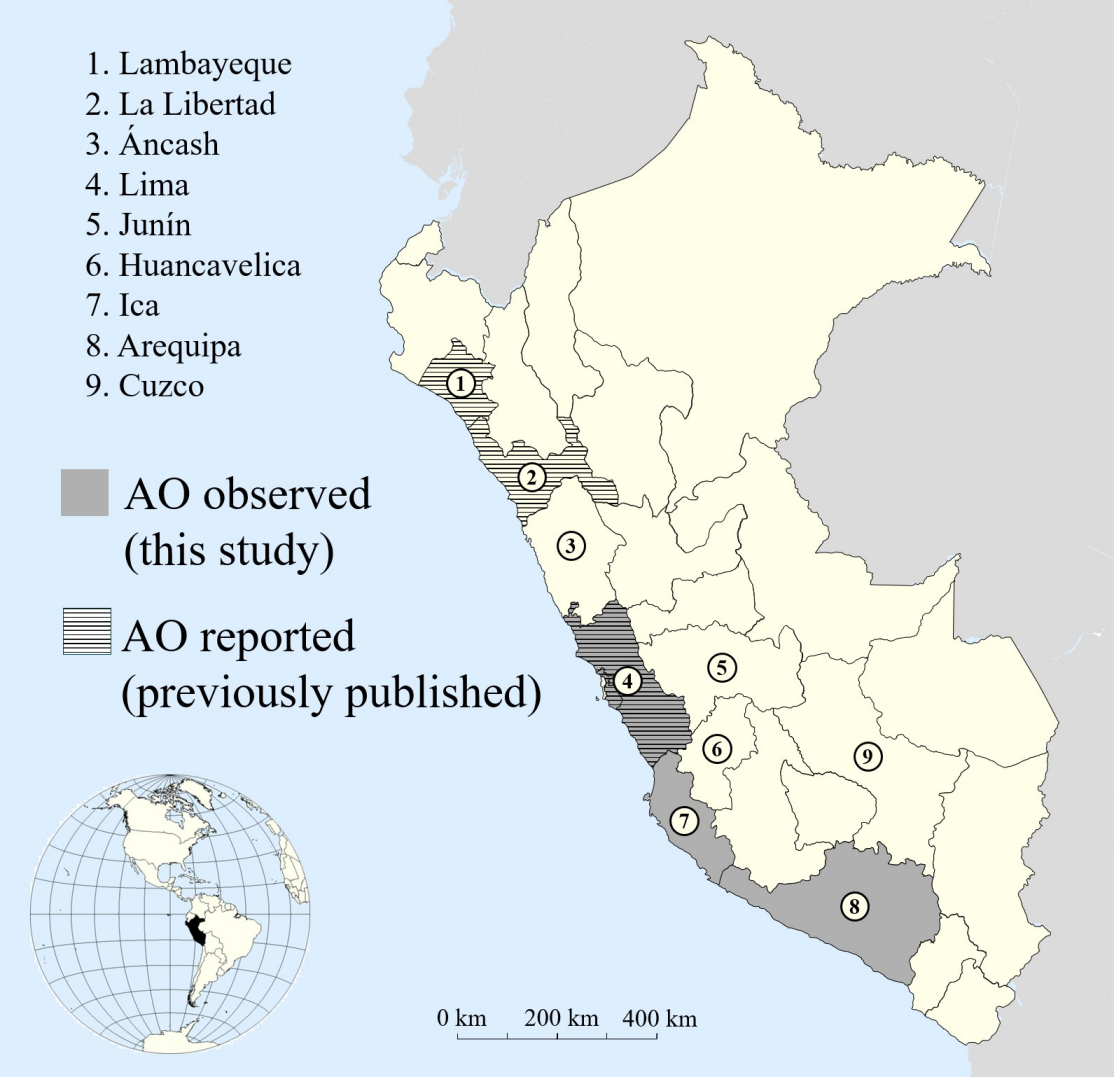
Geographical distribution of atlas occipitalization (AO) across Peru. Crania examined for this study were from regions 2–9, with AO observed in regions 4, 7, and 8 (in gray). Previous reports of AO (in stripes) come from Verano [4] in region 1 and Barnes [3] in regions 2 and 4. This figure is a derivative of “Blank map of South America” by Camilo Sanchez and “Western Hemisphere of Earth (Lambert Azimuthal projection)” by Sean Baker, which are licensed under CC BY 3.0 and 2.0, respectively.

Data shows the number of crania with and without AO by region, percentage of crania with and without AO as a proportion of each regional total, and total sample size from each region as a percentage of total sample size. Region numbers correspond to the legend in Fig 1. Regions represent the broadest level of geographical classification recorded by the NMNH and are consolidated from provincial-, district-, or site-level locations when necessary.

Crania were categorized into age classes of infant/child (under 12 years old), adolescent (12–20 years old), and adult (over 20 years old) following standard procedures of age estimation based on dental eruption and cranial suture fusion [41, 42]. Crania with any deciduous teeth and unfused sutures were classified as children, those with only permanent dentition in which the third molar had not completed eruption and unfused sutures were classified as adolescent, and those that exhibited third molar eruption and significant fusion of cranial sutures were classified as adults. Crania too fragmented for reliable classification were categorized as indeterminate. Sex was recorded based on labels on adult crania, which correspond to previous determinations entered into museum records. Sex estimations were made by Hrdlička using his standards of the time [43] and his experiences with NMNH collections and collecting in Peru [David Hunt, personal communication, August 2020]. The sex of crania without labels—mostly subadults or excessively fragmented—were categorized as indeterminate.

All crania were visually examined for the presence of ACM. Each cranium was classified as unmodified or modified based on the shape of the cranial profile. Modification was categorized according to a three-type system originally developed for populations in north-central Peru [44]:

1. Posterior flattening is characterized by a flattened occipital bone, widened parietal region, and unaffected frontal bone consistent with cradle practices—the use of devices with a backboard to protect and carry babies [45].
2. Bilobed modification is characterized by flattened occipital and frontal regions, formation of two “lobes” in the parietal region, and depressed sagittal suture. This type of ACM was probably produced using a headdress with a band along the sagittal suture [46, 47].
3. Circumferential modification is characterized by a narrow, elongated cranial shape created by binding the cranium around its circumference with cloth or other materials [44].

Examples of each ACM type are shown in S1 Fig, Panel 1. If ACM was identified in a fragmentary cranium, ACM type was classified as indeterminate.

AO was recorded as absent or present. If present, it was categorized as partial or complete (S1 Fig, panel 2). Complete fusion of the atlas indicates full fusion of the anterior and posterior arch to the occipital bone around the foramen magnum, whereas partial fusion is incomplete in either region [1]. In crania with AO, the presence of spina bifida and fusion of the transverse processes (absent, unilateral, bilateral) to the occipital bone were recorded using diagnostic features from Senator and Gronkiewicz [1] (S1 Fig, panels 3 and 4). Crania were carefully assessed to ensure that there were no signs of fragmentation around the foramen magnum that would indicate that the atlas had been detached postmortem.

Statistical analyses were performed using MS Excel (Microsoft Office 2016) and RStudio (v3.6.1). Chi-square tests were used to assess the relationship between AO and ACM presence, ACM type, geographic region, and sex. The threshold for significance in all tests was α = 0.05. Following tests that produced a significant result, adjusted Pearson residuals were used to identify the combination of variables that differed significantly from expected frequencies. If an assessment of sex, age, presence of ACM, spina bifida, or fusion of the transverse processes could not be made due to fragmentation, the individual was excluded from that analysis.

## Results

Out of a total of 608 crania, eleven cases of AO were observed in three coastal regions—Arequipa, Ica, and Lima—at an overall frequency of 1.8% (Fig 1 and Table 1). AO was observed in eight of the 234 crania (3.4%) estimated as male, none of the 262 crania estimated as female, and three of the 109 crania of indeterminate sex (S1 Table). The frequency of AO varied significantly by sex, both when including crania of indeterminate sex (χ^2^ = 8.7, p = 0.01) and excluding them (χ^2^ = 9.1, p = 0.003). AO occurred less than expected in females (*r*_P_ = -2.1) and more than expected in males (*r*_P_ = 2.2). All cases of AO were observed in adult crania. Summaries of the composition of the sample by sex and age can be found in S1 Table.

### AO and artificial cranial modification

ACM was observed in each of the eight regions surveyed: La Libertad, Áncash, Lima, Junín, Huancavelica, Ica, Arequipa, and Cuzco. Out of the total sample of 608 crania, 381 (62.9%) were modified (Table 2). ACM was observed in eight of the nine (88.9%) crania with AO that could be assessed for the presence of ACM and in 62.5% of the crania without AO (Table 2). The neurocrania of two crania that exhibited AO were too fractured to determine the presence or absence of ACM and were excluded from this statistical analysis. All cases of AO in association with ACM were in crania with posterior or indeterminate modification. There was not a significant association between the presence of ACM and presence of AO (χ^2^ = 2.7, p = 0.1). Although there was a significant association between ACM type and presence of AO (χ^2^ = 22.4, p<0.001), this relationship is driven by the high number of fragmented crania with AO that were classified as indeterminate ACM (*r*_P_ = 4.5) (Table 2).

**Table 2:** Artificial cranial modification (ACM) observed in the sample.

ACM Type	Without AO		With AO		Total	
	n	%	n	%	n	%
Unmodified	224	37.5	1	11.1	225	37.1
Total Modified	373	62.5	8	88.9	381	62.9
Posterior	274	45.9	4	50.0	278	45.9
Bilobed	58	9.7	0	0.0	58	9.6
Circumferential	17	2.9	0	0.0	17	2.8
Indeterminate	24	4.0	4	50.0	28	4.6

Data are shown as the number and percentages of crania with and without ACM in the sample as a whole and in the sample subsets that do and do not exhibit atlas occipitalization (AO). ACM is divided by type.

### Regional variation in AO

The frequency of crania with AO differed significantly by region (χ^2^ = 26.0, p<0.001). AO occurred at frequencies of 7.9% in Arequipa, 4.4% in Ica, and 1.0% in Lima (Table 1, Fig 1). The crania with AO from Arequipa come from sites near the present-day city of Chaviña in the Acarí Valley and the port of Lomas, located around 25 kilometers from the same valley. The crania with AO from Ica come from sites in the Nazca region, including Coyungo. The cranium with AO from Lima comes from the Pasamayo site, located near the mouth of the Chancay River. AO occurred at a significantly higher rate in Arequipa compared to that of the sample overall (*r*_P_ = 4.0). AO was not observed in Áncash, Cuzco, Huancavelica, Junín, or La Libertad, but the samplings from the first four regions (n = 2, n = 5, n = 10, n = 29, respectively) are too small individually to expect instances of AO, given the average rate of AO is 1.8% across all regions.

### Morphological variation in AO

Completeness of occipitalization could be determined for 10 of the 11 crania with AO. Among these 10 crania, 90.0% exhibited partial AO (S2 Table). Spina bifida was observed in one of the 6 crania (14.3%) that were not too fragmented to assess. AO occurred with fused transverse processes in 6 out of 10 crania (60.0%). The transverse processes of the atlas were unilaterally fused in two (20.0%) and bilaterally fused in four (40.0%) of these crania (S2 Table).

Additional vertebral anomalies were observed in one of the 11 crania with AO. The cranium of P293952-0 is asymmetrically positioned to the left over the atlas and foramen magnum. The region, age, sex, ACM type, AO fusion type, presence of spina bifida, and fusion of the transverse processes determinations for each cranium with AO are available in S3 Table.

## Discussion

The frequency of AO in this sample (1.8%) is consistent with figures already published, falling slightly above the rate of 1% calculated for a broad group of prehistoric populations [5] but within the range of 0.03–3.6% reported in anatomical collections of present-day populations [13, 14]. However, this frequency cannot be compared to any values specific to Peruvian populations, past or present, as we are not aware of any population-level studies of AO in Peru that have been published. AO occurs at a significantly higher rates in males than in females in our sample, which is consistent with Gholve et al. (2007) [28], although no difference between sexes has been reported elsewhere [19, 21].

### AO and artificial cranial modification

Humans on every inhabited continent have practiced ACM [32], with the earliest cases reaching back tens of thousands of years [44, 48, 49]. ACM was particularly widespread among populations in the Andean region of South America [44, 47, 50]. While ACM does not usually impact health negatively [5, 51] or lead to a reduction in brain volume, it is associated with an increase in the number of sutural bones [52] and changes in facial morphology [53]. If modification is performed over a short time period or by using particularly intense force, some researchers have proposed that ACM can lead to seizures or epilepsy [54], infections, necrosis, or intracranial hypertension [32].

We hypothesized that ACM could induce or influence some form of atlas fusion due to mechanical stress on the cervical region. However, we did not find evidence of a relationship between ACM of any type and AO presence in our sample, suggesting that AO cannot confidently be added to the list of physiological effects of ACM in this sample of pre-Hispanic Peruvians. A potential relationship between ACM and AO might be obscured by the estimated 200-year temporal spread of our sample, as the prevalence of ACM is known to have fluctuated quite drastically over a similar time span among Quechua and Aymara groups who inhabited the Colca Valley in Arequipa [34]. In addition, the bones of the cranial vault, base, and face respond to ACM to a variable and population-dependent extent [37, 38]. Accordingly, the cervical regions in the populations represented in this sample may not respond to ACM in the form of AO. More likely, however, is that the ACM techniques in our sample did not induce mechanical stress on the atlas to the extent hypothesized.

It is difficult to identify the exact apparatuses that created the variety of ACM types in Peru [32], but Allison et al. [47] have published a study that attempts to link cranial shapes to the modification apparatuses that may have produced them. A cradleboard apparatus [47] would completely immobilize an infant’s head under wraps and cause posterior flattening of the cranium, which is the only type of ACM identifiable in crania with AO in this study (n = 4). However, this finding is likely explained by sample bias, as crania with posterior modification represent 73.0% of the modified crania (versus 15.2% bilobed and 4.5% circumferential) in our total sample. The apparatuses that correspond to the other ACM types seen in our sample are mostly cushions, cloth head bindings, and wool or reed pads [47], which suggests they did not add much additional weight to a child’s head. Other reconstructions of apparatuses from Peru during this time period [46, 55] also indicate that the mechanical stress on wearers was not excessive in terms of weight or restriction of movement.

### Regional variation in AO

To our knowledge, cases of AO in Peru have only been reported in regions along the northern and central coasts. AO has been documented in an archaeological site in Lambayeque dated to around 300 CE [4] as well as in NMNH collections from Lima and La Libertad [3]. Here, we report cases of AO in the southern coastal regions of Ica and Arequipa for the first time, as well as in Lima (Fig 1).

We found a correlation between AO and region, with many more occurrences of AO in Arequipa than the sample as a whole. Although AO occurs at a very low frequency in the sample overall (1.8%), the rate of AO is over four times as high in Arequipa (7.9%). This figure is well above the highest previously-published frequency of 3.6% [14]. AO was also observed in crania from Coyungo in Ica, sites in the Nazca region of Ica, and Pasamayo in Lima. These sites lie along the southern coast, suggesting that AO may be particularly common in this area. Although AO seems to occur exclusively in coastal Peru in our sample, the samples from highland regions of Peru (Áncash, Junín, Huancavelica, and Cuzco) are too small (n = 46) in comparison to the samples from regions with coastlines (n = 562) to conclude that AO did not occur in these regions, given its average frequency of only 1.8% across all regions.

Sample bias toward coastal regions may explain the higher number of cases in coastal regions, but the sample subsets from both La Libertad and Lima also include a few eastern sites located within the geographic bounds of the Andes, which may counterbalance this effect [56]. In addition, rates of AO were significantly higher only in Arequipa, not in all coastal regions, which suggests that sample bias toward coastal regions is not responsible for the identification of Arequipa as a region where AO is particularly common.

In this sample, AO occurs in regions along the southern coast of Peru. This geographic pattern raises questions about the hereditary and environmental factors that are potentially implicated in the etiology of AO in pre-Hispanic Peruvian populations. Kalla et al. [17] and Plischuk [57] have both proposed that AO can be inherited according to a complex inheritance pattern. Certain genetic disorders and anomalies occur at elevated rates among people living in relatively small, isolated populations [58–60], and during the Late Intermediate Period (1000–1400 C.E.) in Peru, many southern populations lived in such groups [61, 62]. AO might thus occur at elevated rates in these regions if families in locally-restricted cultural groups carried specific genetic variations that increased their likelihood of developing AO.

Environmental factors related to diet and disease may also shape the geographic distribution of AO in our sample, although their influence is more speculative. Both poor nutrition [8] and disease [8, 19] have been proposed as factors that can predispose a fetus toward AO [8, 11, 27]. Coastal Peruvian populations ate less varied diets and were more sedentary [63], and some Peruvian groups who lived farther inland in coastal regions were largely dependent on maize [64, 65] and may have developed nutritional deficiencies [66, 67] that could contribute to the development of AO. However, the proximity of many more coastal groups in Arequipa, Ica, and Lima to a rich variety of supplementary crops [64, 68, 69] and protein sources [66, 70, 71] makes it less likely that maize-related malnutrition was universal among them. Ultimately, any firm conclusions about the diets of the individuals in our sample would require stable isotopic analyses, given the large temporal spread and lack of geographic specificity within each region.

In addition to malnutrition, AO has been linked to degenerative conditions like osteoarthritis [11] and infectious diseases like tuberculosis and syphilis [27]. Evidence from the north coast of Peru indicates that degenerative joint disease was one of the most common pathological conditions [72]. Farther south, degenerative diseases of the spine were also the most common spinal anomaly in an analysis of remains from the Valley of Ica in Peru and Valley of Azapa in Chile. Approximately 30% of individuals showed signs of cervical and lumbar osteoarthritis [73], suggesting that degenerative diseases were relatively common among some southern Peruvian populations and, crucially, affected the cervical region. These conditions, however, were unobservable in this study due to the absence of postcrania associated with the crania in our sample.

Cultural activity patterns could also be linked to degenerative changes to the cervical vertebrae, particularly given that all cases of AO in this sample with firm age designations are adults. Carrying a *capacho* or tumpline, a basket used in the Andean region to transport heavy loads [73], is one potential cause of these degenerative changes. *Capacho* straps rest on the wearer’s forehead, so the cervical vertebrae bear most of the mechanical force generated by wearing the basket. The use of a *capacho* might generate the types of degenerative cervical changes that could lead to AO, but its use among the populations in this sample would be difficult to ascertain due to the lack of material culture associated with the remains.

The few existing paleopathological studies of Andean South America emphasize that pathological conditions were more prevalent in sedentary populations [74] such as those on the coast of Peru [63]. Tuberculosis was most common in the far south of pre-Hispanic Peru [74–76], where we identified the highest rates of AO in our sample. Several probable cases of treponemal disease have also been reported in Peru [77, 78], but evidence of venereal syphilis in Peru is less convincing [63]. The lack of associated postcranial elements in our sample significantly limits our ability to examine potential links between these diseases and crania with AO, but in future research, ancient DNA analyses could provide insights into connections between tuberculosis [75, 76, 79, 80] or syphilis [81, 82] and AO through identification of the causal bacteria.

### Morphological variation in AO

Our assessment of morphological features associated with AO builds on many isolated reports in medical and anthropological literature. With a quantitative approach and a more robust sample size than previous studies, our findings more fully characterize the range of variation in AO. Partial AO is frequently cited as being more common than complete AO [1, 19], but there are few statistics on the relative prevalence of each type based on large sample sizes [83]. Results from our sample support a higher prevalence of partial AO, as 90.0% of the crania with AO exhibited partial AO. Fused transverse processes occurred at a rate of 60.0% among crania with AO, which is higher than the 10.0% reported for a sample of crania with AO from Northern India [84]. Differences in the types and influence of etiological factors that give rise to AO in these populations may explain the higher rate in this sample.

Spina bifida is a type of neural tube disorder that occurs when the fetal spinal column does not fully close before birth, which can cause a variety of physical symptoms. Osteologically, spina bifida is defined as the failure of the two lamina of a vertebra to unite and form a continuous bony arch [85]. Neural tube disorders are considered to have a strong genetic component [86], but they do not have a single cause and involve a number of dietary factors that may be associated with AO, such as an elevated glycemic index [87] and amino acid deficiencies [88, 89]. Spina bifida of the posterior arch of the atlas frequently occurs alongside AO in this sample, at a rate (14.3%) roughly two times as great as that among contemporary Peruvian populations (6.1%) [90]. However, spina bifida has been reported at rates as high as 70% in some archaeological sites from the Ica and Azapa Valleys in Peru and Chile [73], indicating that the observed rate in this sample falls well within a reasonable range. Ultimately, the lack of associated postcrania makes it more difficult to characterize population-specific rates of spina bifida among individuals with and without AO in this sample.

One cranium with AO (P293952-0) also exhibits characteristics of torticollis, a congenital or acquired anomaly of the cervical region that causes the head to tilt to one side due to an abnormally rotated cervical spine [91]. Torticollis usually arises from muscular or vertebral anomalies in the craniocervical region [91], so it may occur alongside or result from the presence of AO. This individual may have experienced additional symptoms and restriction of head movement related to torticollis than individuals with AO only.

## Conclusions

This study, in conjunction with those published elsewhere [3, 4], documents the presence of AO in human populations along the central and southern Peruvian coast ca. 1300–1500 CE. We hypothesized that AO, which has been associated with biological factors such as heredity, nutrition, and disease, may also be associated with ACM, a cultural practice. However, we did not find evidence of a significant association between ACM and AO, a result which may be due to low levels of mechanical stress associated with the ACM apparatuses used to produce the types of ACM in our sample.

Across the geographic area that we sampled, we observed significantly elevated rates of AO in Arequipa, a southern coastal region, which invites a discussion of AO’s higher prevalence in this area. Due to the lack of geographic and temporal specificity in NMNH records, however, our results should be interpreted only on a very broad scale of time and geographic location. Hereditary, dietary, and epidemiological conditions should be further explored as factors shaping the distribution of AO in Peru in order to clarify some of the complex and poorly-understood causes of a spinal anomaly that continues to affect diverse populations across the globe.

## Supporting information

S1 Fig Morphological features of crania assessed in this study. Panel 1 shows the five categories of artificial cranial modification (ACM): unmodified (1a), posterior flattening (1b), bilobed (1c), circumferential (1d), and indeterminate (1e). Panel 2 shows examples of an occipital bone with no occipitalized atlas present (2a), occipitalized atlas with partial fusion in both the anterior and posterior arch (2b), and occipitalized atlas that is completely fused all the way around the foramen magnum. Panel 3 shows an occipitalized atlas that does not exhibit spina bifida (3a), and an occipitalized atlas that does exhibit spina bifida (3b). Panel 4 shows examples of unfused transverse processes (4a), unilateral fusion (4b), and bilateral fusion (4c).
(TIF)

S1 Table Sex and age composition of the sample. Data are shown as raw counts and percentages for the sample as a whole and for the sample subsets that do and do not exhibit atlas occipitalization (AO).
(DOCX)

S2 Table Morphological features associated with atlas occipitalization (AO). Data are shown as raw counts and percentages of the total number of crania with AO.
(DOCX)

S3 Table Characteristics of crania that exhibit atlas occipitalization (AO). Data include region, age (A = Adult), sex (M = Male, F = Female, UK = Unknown), completeness of AO, presence of spina bifida, and description of fusion of transverse processes. Starred crania are fragmentary, and unobservable attributes are marked with an “X.”
(DOCX)

S1 File Table of complete survey data of crania sample. All remains are held at the Museum Support Center in Suitland, Maryland, which is a storage facility for the National Museum of Natural History (NMNH or USNM), Smithsonian Institution, located in Washington, D.C.
(XLSX)
