# Supplementary figures and images for: Population-level assessment of atlas occipitalization in artificially modified crania from pre-Hispanic Peru
Source: PLoS One. 2020 Sep 24;15(9):e0239600. doi: 10.1371/journal.pone.0239600 (PMC7514022; doi:10.1371/journal.pone.0239600)

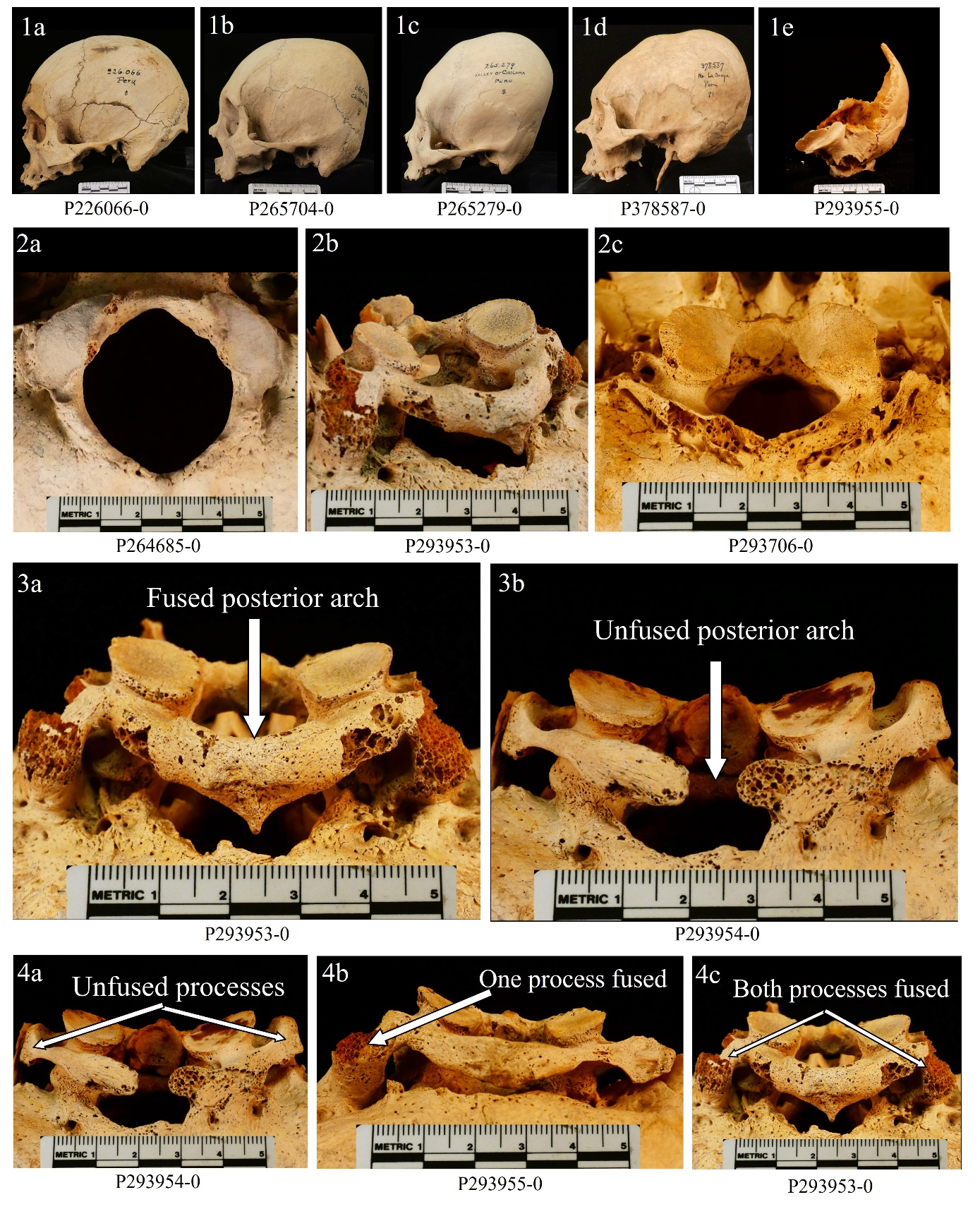

Supplement: S1 Fig — Panel 1 shows the five categories of artificial cranial modification (ACM): unmodified (1a), posterior flattening (1b), bilobed (1c), circumferential (1d), and indeterminate (1e). Panel 2 shows examples of an occipital bone with no occipitalized atlas present (2a), occipitalized atlas with partial fusion in both the anterior and posterior arch (2b), and occipitalized atlas that is completely fused all the way around the foramen magnum. Panel 3 shows an occipitalized atlas that does not exhibit spina bifida (3a), and an occipitalized atlas that does exhibit spina bifida (3b). Panel 4 shows examples of unfused transverse processes (4a), unilateral fusion (4b), and bilateral fusion (4c). (TIF) [file pone.0239600.s001.tif]
