# Supplementary material for: Population-level assessment of atlas occipitalization in artificially modified crania from pre-Hispanic Peru
Source: PLoS One. 2020 Sep 24;15(9):e0239600. doi: 10.1371/journal.pone.0239600 (PMC7514022; doi:10.1371/journal.pone.0239600)
Supplement: S1 Table — Data are shown as raw counts and percentages for the sample as a whole and for the sample subsets that do and do not exhibit atlas occipitalization (AO). (DOCX) [file pone.0239600.s002.docx]

|  | | Without AO | | With AO | | Total | |
| --- | --- | --- | --- | --- | --- | --- | --- |
|  |  | n | % | n | % | n | % |
| Sex | Female | 262 | 43.9 | 0 | 0.0 | 262 | 43.1 |
|  | Male | 226 | 37.9 | 8 | 72.7 | 234 | 38.5 |
|  | Indeterminate | 109 | 18.3 | 3 | 27.3 | 112 | 18.4 |
| Age | Adult | 498 | 83.4 | 7 | 63.6 | 505 | 83.1 |
|  | Adolescent | 64 | 10.7 | 0 | 0.0 | 64 | 10.5 |
|  | Infant/Child | 7 | 1.2 | 0 | 0.0 | 7 | 1.2 |
|  | Indeterminate | 28 | 4.7 | 4 | 36.4 | 32 | 5.3 |

**S1 Table. Sex and age composition of the sample.** Data are shown as raw counts and percentages for the sample as a whole and for the sample subsets that do and do not exhibit atlas occipitalization (AO).
