# Supplementary material for: Population-level assessment of atlas occipitalization in artificially modified crania from pre-Hispanic Peru
Source: PLoS One. 2020 Sep 24;15(9):e0239600. doi: 10.1371/journal.pone.0239600 (PMC7514022; doi:10.1371/journal.pone.0239600)
Supplement: S2 Table — Data are shown as raw counts and percentages of the total number of crania with AO. (DOCX) [file pone.0239600.s003.docx]

**S2 Table. Morphological features associated with atlas occipitalization (AO).** Data are shown as raw counts and percentages of the total number of crania with AO.

| Morphological Feature | Classification | n | % |
| --- | --- | --- | --- |
| Completeness of occipitalization | Partial | 9 | 90.0 |
|  | Complete | 1 | 10.0 |
| Spina bifida | Present | 1 | 14.3 |
|  | Absent | 6 | 85.7 |
| Fused transverse processes | Unfused | 4 | 40.0 |
|  | Unilateral | 2 | 20.0 |
|  | Bilateral | 4 | 40.0 |
