# Supplementary material for: Population-level assessment of atlas occipitalization in artificially modified crania from pre-Hispanic Peru
Source: PLoS One. 2020 Sep 24;15(9):e0239600. doi: 10.1371/journal.pone.0239600 (PMC7514022; doi:10.1371/journal.pone.0239600)
Supplement: S3 Table — Data include region, age (A = Adult), sex (M = Male, F = Female, UK = Unknown), completeness of AO, presence of spina bifida, and description of fusion of transverse processes. Starred crania are fragmentary, and unobservable attributes are marked with an “X.” (DOCX) [file pone.0239600.s004.docx]

**S3 Table. Characteristics of crania that exhibit atlas occipitalization (AO).** Data include region, age category, sex, completeness of AO, presence of spina bifida, and description of fusion of transverse processes. Starred crania are fragmentary, and unobservable attributes are marked with an “X.”

| **ID** | **Location in Peru** | **Age** | **Sex** | **ACM** | **ACM Type** |  | **AO Completeness** | **Fused Transverse Processes** | **Spina Bifida** | **Notes** |
| --- | --- | --- | --- | --- | --- | --- | --- | --- | --- | --- |
| ***P293952-0**** | Ica (?) | Adult | M | Absent | n/a |  | Partial | Bilateral | 0 | Cranial fragment. Lomas, Peru. Location is listed as Ica (?), but crania included in Arequipa in all analyses |
| ***P293953-0**** | Ica (?) | Adult | M | Present | Undefined |  | Partial | Bilateral | 0 | Cranial fragment. Lomas, Peru. Location is listed as Ica (?), but crania included in Arequipa in all analyses |
| ***P293954-0**** | Ica (?) | X | M | Present | Undefined |  | Partial | Absent | 1 | Cranial fragment. Lomas, Peru. Location is listed as Ica (?), but crania included in Arequipa in all analyses |
| ***P293955-0**** | Ica (?) | X | M | Present | Undefined |  | Partial | Unilateral | 0 | Cranial fragment. Lomas, Peru. Location is listed as Ica (?), but crania included in Arequipa in all analyses |
| P293706-0 | Arequipa Chavina | Adult | M | Present | Posterior |  | Complete | Bilateral | 0 |  |
| P293244-0 | Ica: Nazca Region | Adult | M | Present | Posterior |  | Partial | Absent | X | Posterior arch of atlas partially fragmented |
| ***P293255-0**** | Ica: Nazca Region | Adult | UK | Present | Undefined |  | Partial | Absent | 0 | Cranial fragment |
| ***P293260-0**** | Ica: Nazca Region | Adult | M | Present | Posterior |  | Partial | Absent | X | Cranial fragment. Posterior arch of atlas partially fragmented |
| P379278-0 | Lima, Pasamayo Site | Adult | M | Present | Posterior |  | Partial | Bilateral | X | Posterior arch of atlas partially fragmented |
| ***P293965-0**** | Ica (?) | X | X | X | X |  | X | X | X | Basicranial fragment, posterior arch of atlas partially fragmented. Lomas, Peru. Location is listed as Ica (?), but crania included in Arequipa in all analyses |
| ***P293305-0**** | Ica | X | X | X | X |  | Partial | Unilateral | 0 | Basicranial fragment |

All crania from Peru: Ica (?) come from the port of Lomas, which is located in Arequipa and was erroneously attributed to Ica in NMNH records.
